# Mapping out bare-nosed wombat (*Vombatus ursinus*) burrows with the use of a drone

**DOI:** 10.1186/s12898-019-0257-5

**Published:** 2019-09-18

**Authors:** Julie M. Old, Simon H. Lin, Michael J. M. Franklin

**Affiliations:** School of Science and Health, Western Sydney University, Hawkesbury Campus, Locked Bag 1797, Penrith, NSW 2751 Australia

**Keywords:** Unmanned aerial vehicle, Aerial survey, Wombat burrow survey, Wildlife monitoring

## Abstract

**Background:**

Wombats are large, nocturnal herbivores that build burrows in a variety of habitats, including grassland communities, and can come into conflict with people. Counting the number of active burrows provides information on the local distribution and abundance of wombats and could prove to be an important management tool to monitor population numbers over time. We compared traditional ground surveys and a new method employing drones, to determine if drones could be used to effectively identify and monitor bare-nosed wombat burrows.

**Results:**

We surveyed burrows using both methods in eight 5-ha transects in grassland, that was interspersed with patches of tussock grassland. Ground surveys were conducted by systematically walking transects and searching for burrows. Drone surveys involved programming flights over transects to capture multiple images, from which an orthomosaic image of each transect was produced. These were subsequently viewed using ArcMap to detect burrows. A total of 204 individual burrows were recorded by drone and/or ground survey methods. In grassland, the methods were equally effective in terms of the numbers of burrows detected in transects. In the smaller areas of tussock grassland, ground surveys detected significantly more burrows, because burrow openings were obscured in orthomosaic images by overhanging grasses. There was agreement between the methods as to whether burrows were potentially active or inactive for most burrows in both vegetation communities. However, image interpretation tended to classify grassland burrows as potentially active. Overall time taken to conduct surveys was similar for both methods, but ground surveys utilised three observers and more time in the field.

**Conclusions:**

Drones provide an effective means to survey bare-nosed wombat burrows that are visible from the air, particularly in areas not accessible to observers and vehicles. Furthermore, drones provide alternative options for monitoring burrows at the landscape level, and for monitoring wombat populations based on observable changes in burrow appearance over time.

## Background

Wombats are large herbivorous marsupials, native to Australia, that reside underground in burrows during the day [26]. Bare-nosed wombats (*Vombatus ursinus*) are distributed in south-eastern Australia from southern Queensland to eastern South Australia, including Tasmania and some islands in Bass Strait [26], and are listed as least concern on the IUCN red list [40]. Southern hairy-nosed wombats (*Lasiorhinus latifrons*) are distributed in mainland areas of southern Australia [41] and listed as near threatened [48]. However, despite both species being protected, and numbers of bare-nosed wombats unknown, permits to reduce numbers of both species can be obtained when they come into conflict with people. Conflicts can occur with farmers where wombats may build burrows and undermine fences, buildings and farming equipment. The burrows built by wombats may pose physical hazards to horses and cattle, and wombats can also cause significant damage to vehicles when hit. Determining the population numbers of wombats is therefore important both ecologically and to aid in reducing the number of human–wildlife conflicts.

Surveying the number of wombat burrows is non-invasive and can provide valuable distribution data required for wombat management and conservation purposes [5, 34, 35]. However, counting wombat burrows can be difficult and very time consuming, especially in areas which cannot be accessed by foot or vehicle, and can pose safety and accessibility issues.

Recent advances in technology are supporting a wide variety of ecological and wildlife field research. High-resolution spatial imagery captured by unmanned aerial vehicles (hereafter, drones) can facilitate detection and monitoring of invasive plant species in vulnerable habitats, thus aiding management through identification of new invasions [2]. Drones can be used to assess tree hazards in urban environs [19], and habitat composition [13]. Drones have also been used to monitor agricultural performance [49], and resolve human–elephant conflicts by removing elephants from corn fields and settlements, hence reducing risks to elephants and people as well as reducing crop loses [15].

Drones can also be used to monitor wildlife and the development of drone-based survey methods is an active area of research. Drones are a cost-effective substitute for manned aircraft to survey animals that are most effectively surveyed from above, such as the Nile crocodile (*Crocodylus niloticus*) [11]. Drones have been used to conduct aerial surveys of gray seals (*Halichoerus grypus*), with no apparent effects on the behaviour of seals [3]. There were no differences in abundance or changes in orientation or posture of imaged seals, hence it was recommended as a best practice method to survey gray seal colonies. Drone surveys of nesting lesser snow geese (*Anser caerulescens caerulescens*) resulted in some disturbance, but it was regarded as minimal [4]. In Australia, drones have been used to obtain accurate population estimates of waterbirds, and when compared to traditional manned aerial surveys, had fewer logistical issues [24].

The use of drones with on-board digital cameras to survey wombat habitat may identify bare-nosed wombat burrows more efficiently than walking or driving surveys. Furthermore, drones are fairly low cost and improve accessibility [8]. The photographic images taken from a drone can be processed and used in a Geographic Information System (GIS) to aid management through identification of burrow sites, their numbers and information about the surrounding habitat. Hence, this study investigated the effectiveness of using a drone with an on-board camera to survey bare-nosed wombat burrows, and also compared the efficiency and results with those obtained from traditional ground surveys.

## Results

**Fig. 1:**
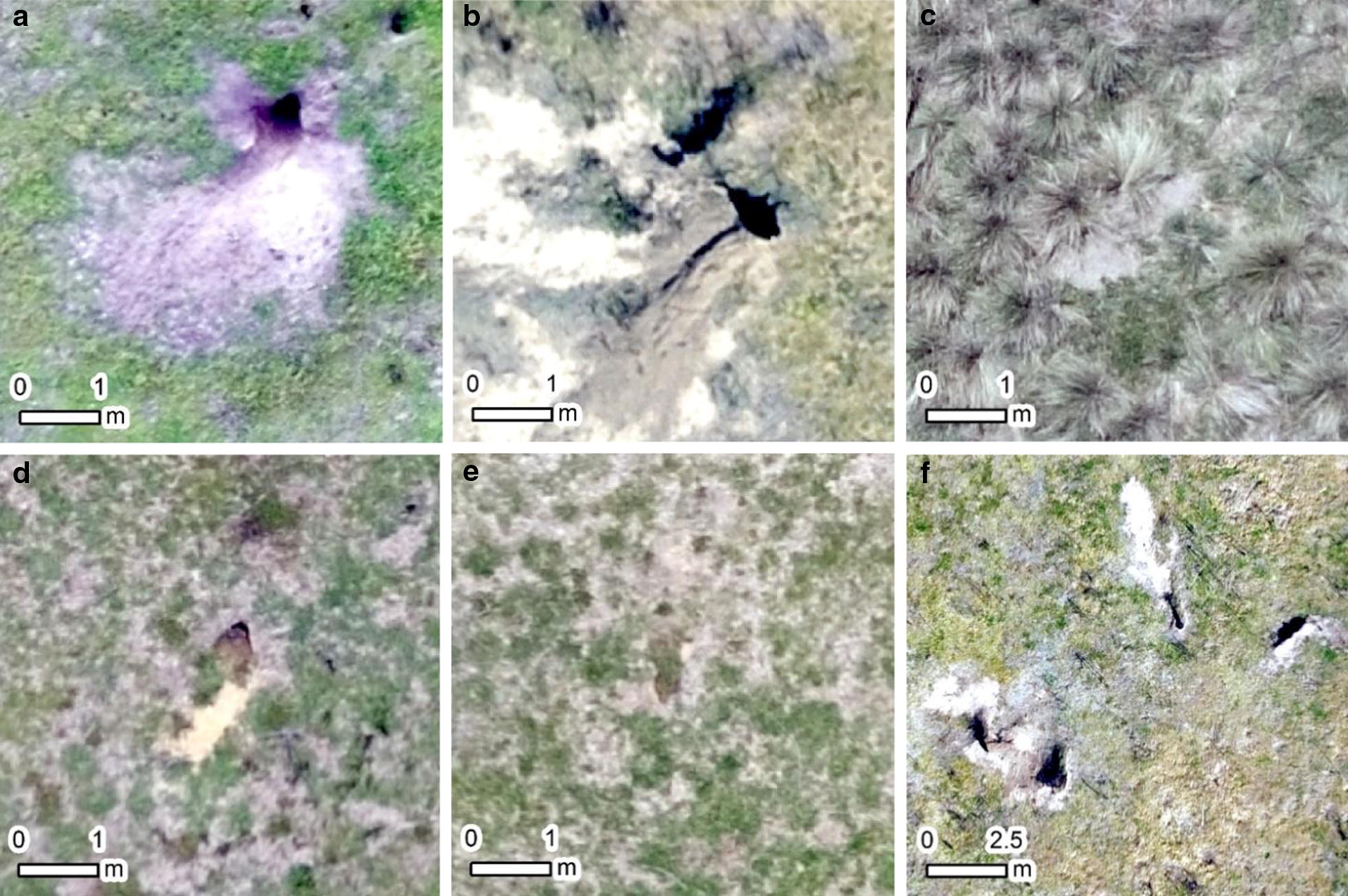
In the image interpretation phase of drone survey, wombat burrows were searched for by viewing orthomosaic images at 1:100 scale, as shown here. Potentially active burrows were clearly evident in grassland (**a**, **b**). In tussock grassland, many burrows had their openings obscured by tussock grasses (**c**). Inactive burrows were also detected by drone and ground survey (**d**), but burrows were harder to discern as apparent time since occupation increased (**e**). Clustered, multiple openings to what was presumably the same burrow were often observed (**f**)

A total of eight transects were surveyed using both ground and drone survey methods. Both methods detected wombat burrows, however the probability of detecting wombat burrows depended on the grassland community in which they occurred, and their likely occupation status. Potentially active burrows in grassland were readily detected by both ground and drone surveys (Fig. 1a, b). In tussock grassland, burrows in orthomosaic images were often obscured by tussock grasses overhanging their entrances, making these burrows more difficult to discern (Fig. 1c). Inactive burrows were also identified using both methods, especially when there was some spoil exposed and a shadow formed by the burrow opening (Fig. 1d), however burrows that appeared to have been inactive for a longer time were less reliably distinguished in orthomosaics (Fig. 1e). The spatial distribution of burrow openings was often clustered in the transects (Fig. 1f).

### Effectiveness of ground and drone surveys

A total of 204 individual burrows were recorded in the eight transects by ground and/or drone survey methods, with the results of survey methods varying considerably according to the type of grassland community in which burrows were located. Totals of 131 and 73 burrows were recorded in grassland and tussock grassland respectively.

**Fig. 2:**
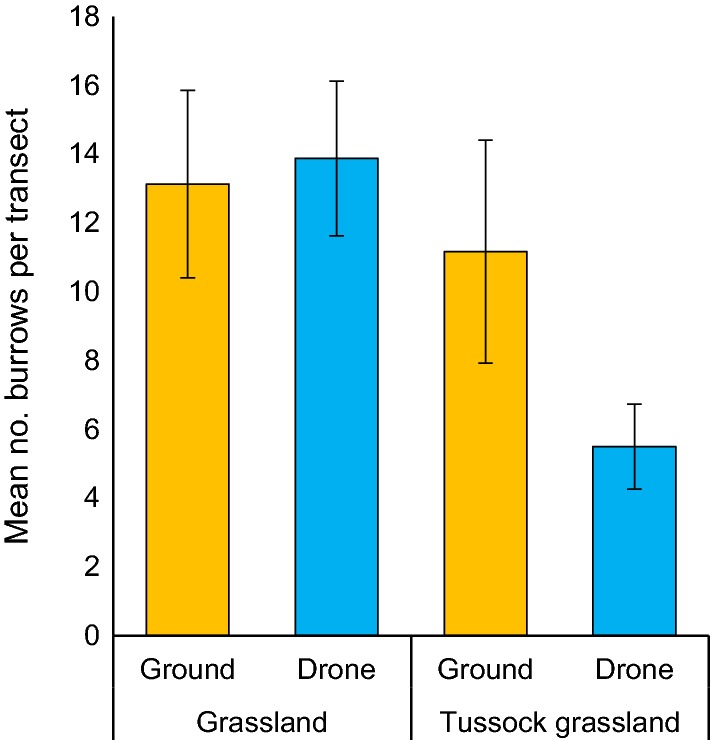
The mean number of burrows (± s.e.) in grassland and tussock grassland areas within transects that were detected by drone survey and ground survey

The two survey methods were in agreement in terms of the number of burrows detected in grassland areas within each transect. In grassland, the mean number of burrows detected per transect was 13.1 by ground surveys and 13.9 by drone surveys (Fig. 2). In this community, the difference in the numbers of burrows recorded by the two survey methods was not significant (*t*_7_ = − 1.34, *p* = 0.22). Fewer individual burrows were detected by the methods on average in tussock grassland (Fig. 2). In areas of transects classified as tussock grassland, an average of 5.7 more burrows (95% CI 0.5, 11.3 burrows) were detected by ground surveys than by drone surveys, hence ground surveys were significantly more effective at detecting burrows in this vegetation type (*t*_5_ = 2.592, *p* = 0.049; Fig. 2).

### Estimation of burrow occupancy status

Of the 112 individual burrows recorded by both survey methods, 76% were detected in grassland and 24% in tussock grassland (Table 1). There was agreement between the methods as to whether burrows were either potentially active or inactive for 68% of burrows in grassland and 59% of burrows in tussock grassland. For the remaining burrows, where the methods disagreed on estimated occupation status, interpretation of orthomosaics appeared to systematically tend towards classifying burrows as potentially active in both vegetation communities (Table 1). In grassland, 15% more burrows (95% CI 4%, 27%) were classified as potentially active using drone methods compared with ground surveys, which was a significant increase (McNemar’s $\chi_{1}^{2}$ = 6.26, *p* = 0.012). Because of the small sample sizes in the tussock grassland categories of Table 1b, McNemar’s Chi-square test was unsuitable, so an exact binomial test was applied [1]. In tussock grassland, there was insufficient evidence to reject the null hypothesis that survey methods were equivalent in relation to classification of occupancy status (*p* = 0.065).

**Table 1:** Extent of agreement between survey methods on estimated burrow occupancy status

	Burrows	Drone survey		Total
		Potentially active	Inactive	
(a) Grassland				
Ground survey	Potentially active	46	7	53
	Inactive	20	12	32
	Total	66	19	85
(b) Tussock grassland				
Ground survey	Potentially active	12	2	14
	Inactive	9	4	13
	Total	21	6	27

Most of the individual burrows observed in the study were detected during both ground and drone surveys of their particular transect. Tables a and b show the numbers of these burrows that were classified as potentially active and/or inactive in each of the two grassy communities. To provide a basis for evaluating agreement of this aspect of the two methods, the marginal proportions in each table were tested for homogeneity using McNemar’s test (see text)

### Resources required for ground and drone surveys

The overall time required to survey a transect was similar for ground and drone methods, but only one person was necessary for the field aspects of drone surveys, and most drone survey time was spent working with imagery at the desktop (Table 2). The time required to establish and set out each transect for ground surveys was very similar to the time taken to plan and configure a drone survey flight over a transect. All aspects of drone surveys were completed by a single skilled operator, and for each transect required on average 15 min of flight time, and ~ 15 min of active involvement to download images from the drone and initiate image processing. Resulting orthomosaic images were visually scanned by the analyst and burrows recorded for 30 min per transect. A total of 60 min per transect was required for these aspects of drone survey.

**Table 2:** Resources required to survey wombat burrows in each transect with ground and drone methods

Method	People		Time (min)			Equipment
	No.	Skill	Field	Desktop	Total	(AUD)
Ground	3	Low/med	57	10	67	GPS × 3 (1200)
Drone	1	High	15	45	60	Drone (2925) Tablet (470) Software (2000)

The time taken to prepare for surveys and to move around the study area was equivalent for the methods and is not accounted for in the field time presented here. The time required at the desktop for the methods involved downloading GPS coordinates for ground surveys, and downloading, processing and interpreting imagery for drone surveys. The costs of essential equipment were for the initial purchase of these items, which may be expected to be used on multiple, varied projects over their service life

Ground surveys were conducted by three observers and were completed in an average of 19 min, so total observer time spent in each transect per survey was 57 min on average (Table 2). It was a requirement that at least one of the ground surveyors was proficient with the technique, so that they could provide training and supervision of the other two participants, to ensure consistency. Approximately 10 min per transect was required to download and store global positioning system (GPS) coordinates of burrow locations collected in the field during ground surveys. While the costs of using staff in the field for ground surveys are potentially much higher compared with drone surveys, the initial costs of the equipment required for the methods was much greater for drone surveys (Table 2).

## Discussion

This study is the first to explore the effectiveness of using a drone to detect bare-nosed wombat burrows and compare this method to traditional ground surveys. We have shown that a drone can be used as an effective research tool to locate bare-nosed wombat burrows in grasslands adjacent to riparian zones where overhanging vegetation, including tree canopies, do not obscure burrow openings from above.

The two survey methods were equally effective at locating burrows in grassland, in that there was no difference in the numbers of burrows detected by the two methods in this vegetation type. However, ground surveys were more effective in the tussock grassland community. In some cases, grass tussocks covered burrows and obscured visibility, and hence were not readily visible in images captured by the drone. Burrows were also more difficult to detect amongst tussock grasses from above during ground surveys; in some cases, burrows were only identified when researchers searched under tussocks to confirm the presence of a suspected burrow. Bare-nosed wombat burrows do occur in a range of habitat types, however they are most prominent in grasslands adjacent to riparian zones [29, 37], such as those we surveyed in this study. Despite our animal ethics approvals limiting the habitats we could survey due to concerns about disturbing nesting birds, future surveys could compare the two methods in other open habitat types. Presumably though areas with trees and shrubs would make it more difficult for the drone to detect wombat burrows, due to the reduced visibility.

One further advantage of using either method to determine differences in abundance and distribution of wombats, based on wombat burrows, is that these survey methods can be undertaken during the day. As wombats are nocturnal [26], techniques used to identify individual wombats, hence population distribution and abundance, generally involve spotlighting [25], and hence impact the wombats to some degree. Surveys conducted by drones may impact some species [4, 16, 27, 46], however by conducting surveys when the wombats are underground in their burrows, it eliminated any potential disturbance to the wombats.

When compared to the overall time taken to conduct ground surveys for bare-nosed wombat burrows, the use of a drone was comparable, however a larger number of researchers (three) was required to conduct ground surveys compared to operating the drone (one). Despite the time taken being similar for both survey methods, the drone survey involved less field time (even including pilot tests to determine optimal flying heights), when compared to ground surveys, but more time at the desktop. Automated detection of objects such as burrows in aerial images has the potential to further reduce processing time (e.g. Chabot and Francis [7]), and the effectiveness of manual or automated detection may be augmented by the use of different sensors. For example, thermal sensors could be used to produce images where burrows might appear prominently [14], because of a likely difference between the burrow temperature and the ground surface temperature at particular times of the day.

With drone methods, there is a trade-off between the spatial extent of a survey and image resolution, which in turn affects burrow visibility. Drone flying height, speed and the amount of image overlap/sidelap are important factors that affect the resolution of images, with greater image resolution effectively reducing the area covered. In particular, reducing the flying height (within safe limits) and increasing image overlap/sidelap can maximise image resolution and therefore burrow visibility. While we optimized surveys via drone flight settings based on the study landscape and environmental conditions, optimization by experimenting with drone settings in preliminary test surveys is recommended for other studies, to account for differing habitat features, drone capabilities, sensors of the digital camera, software, battery life and rapid advances in drone technology and functionality.

Other disadvantages of using the drone to survey for wombat burrows includes the skill level required, such as training required to physically fly the drone and to become familiar with using the software. The initial costs of the drone, accessories and associated software is greater compared to the equipment required for ground surveys. Also, whilst ground surveys can be safely conducted in a fairly wide range of weather conditions, a drone can not be flown when there is any rain, or more than very light wind. Furthermore, patchy clouds may impact burrow visibility in resulting images due to shading effects.

The difference between the number of burrows and burrow openings in terms of counts was unable to be determined from the composite images taken by the drone. Physically checking the burrow entrances is therefore required. Hence other methods to assess burrow occupancy will remain necessary, despite improvements in resolution, because ground-based assessment is not restricted by the angle and position of burrows in the terrain.

When assessing the level of potential burrow occupancy, the drone survey method was limited by the resolution of the orthomosaic images of the transects. The higher proportion of potentially active to inactive burrows with drone surveys is attributable to the classification rules used. For both methods, burrows were classified as potentially active if no vegetation was evident in the exposed soil at the burrow entrance. However, on-ground surveyors may have observed small plants in undisturbed soil at a burrow entrance, and so classified the burrow as inactive, while the same burrow in the corresponding orthomosaic could potentially be ruled as active, because small plants may not be visible. Ground surveyors also reported evidence of burrow inactivity, such as internal burrow collapse, which was not visible in aerial images.

Reliable determination of burrow occupancy status requires effort over time, and methods in addition to short term surveys using either observers or drones are required. Methods to determine wombat burrow occupancy have involved the use of sticks [25] and observation of fresh tracks entering and exiting the burrow, amount of vegetation blocking entrance, and spider webs across entrance [31]. However these methods are limited in their accuracy, as while they provide information that the burrows are in use, they can not confirm they are in use by wombats. The use of digital infra-red camera trapping methods have however proven much more effective in confirming burrow occupancy by wombats [30, 31, 38, 44]. Wombats share and utilize multiple burrows [12, 36], hence while a burrow may appear to have been occupied recently, it may not be occupied on the survey day. Wombats are ecological engineers essential to soil turnover and aeration [30], which can result in habitat creation for a range of other species [18], so it is possible burrows may be occupied by other animals such as foxes and rabbits [29].

Further efforts are also required to more confidently estimate wombat population numbers based on number of active burrows observed, as wombat densities are known to vary in different habitats and food availabilities [6, 10, 25, 22, 37, 43]. Hence, further investigations are required to confirm how many wombat burrows are truly active and inactive, how many wombats use each burrow, and the impact of food availability at this study site at different times. Also, as wombats utilise multiple burrows at different times and share burrows (although usually not at the same time) [37] a promising application of using drones to survey wombat burrows is that of medium and longer-term monitoring of the changes in the distribution and density of burrows. Previously used flight plans may be saved and efficiently used for identical repeat surveys at a later point in time. The use of drones for local monitoring projects has been suggested previously [45]. Given that large areas are also able to be surveyed safely and at low cost [23, 42], drones would be particularly useful for medium and long-term monitoring of wombat burrows. In particular, the capacity to detect new burrows in focal areas would be of value for management and conservation purposes. For example, new burrows detected in a survey may be assumed to have been occupied (active) at some point since the last survey. A drone may also be used to monitor observable changes in burrow appearance, suggesting occupancy or abandonment, thus improve species tracking as described by Hua and Shao [17]. Additionally, the images obtained using a drone can be easily used with GIS software, which enables opportunities for different types of analyses, such as assessment of habitat use.

Currently, the population of bare-nosed wombats is unknown, however wombats are being impacted by sarcoptic mange, road vehicle collisions, habitat destruction, and other human-related impacts [30, 33, 35, 40]. Methods that enable more accurate wombat population numbers to be determined will better inform managers in their decision-making roles in both human–wildlife conflict situations, and conservation. For example, by having more accurate information on abundance estimates of wombats in a certain area, and fluctuations in those estimates, managers will be able to more accurately assess the impacts of environmental factors, such as droughts, on wombat populations. More accurate estimates of wombat abundance will also provide managers with the ability to predict the potential impact of higher or lower numbers of wombats may have in certain areas. For example, obtaining more accurate numbers of a wombat population in farmland could allow managers to better assess the impact wombats are having on the landscape, and the potential for conflicts with farmers and road users.

Satellite imagery has been used successfully to detect southern hairy-nosed wombat (*Lasiorhinus latifrons*) burrows in South Australia [21, 39]. However, it is not currently possible to utilise this technology for bare-nosed wombat burrows, because their burrows are smaller, there is less visible spoil at the entrance of the burrow, and there is a lack of a grazing ‘halo’ that is evident around southern hairy-nosed wombat burrows [39]. Nevertheless, the probable availability of higher resolution satellite imagery in the future may enable burrow surveys for bare-nosed wombats to be conducted in relatively open areas, such as those we surveyed in this study. The costs of drones, cameras, sensors and batteries will also likely decrease in the future, whilst potential accuracy due to increased resolution, and battery storage capabilities will increase, making drones an essential piece of equipment in the field.

## Conclusions

This study explored the capacity of drones to survey and map wombat burrows and compared results with those obtained by ground surveys conducted in the same locations. Only one person was required in the field to successfully operate the drone to capture reliable and accurate survey data. High-resolution orthomosaic images were produced from multiple individual images acquired from the drone, and these were suitable for the purpose of visually detecting wombat burrows at the desktop. The results of this research illustrate that drones are an effective option for surveying wombat burrows and may lead to improved management outcomes for bare-nosed wombats, in undisturbed open areas, and where grassland habitat overlaps with human land use. Specifically, the methods developed and tested in this study could be adopted to monitor bare-nosed wombat population numbers and local distributions, based on burrow counts, on an ongoing-basis. Drones will be a particularly important tool to aid surveys for hard-to-reach populations, and areas that are challenging to access via field vehicles, or on foot. Using drones to survey wombat burrows will also be a particularly useful option when project resources for the deployment of staff in the field are limited. Trialling different types of drones and sensors and surveying different habitat types would be productive areas for future research.

## Methods

### Study site

**Fig. 3:**
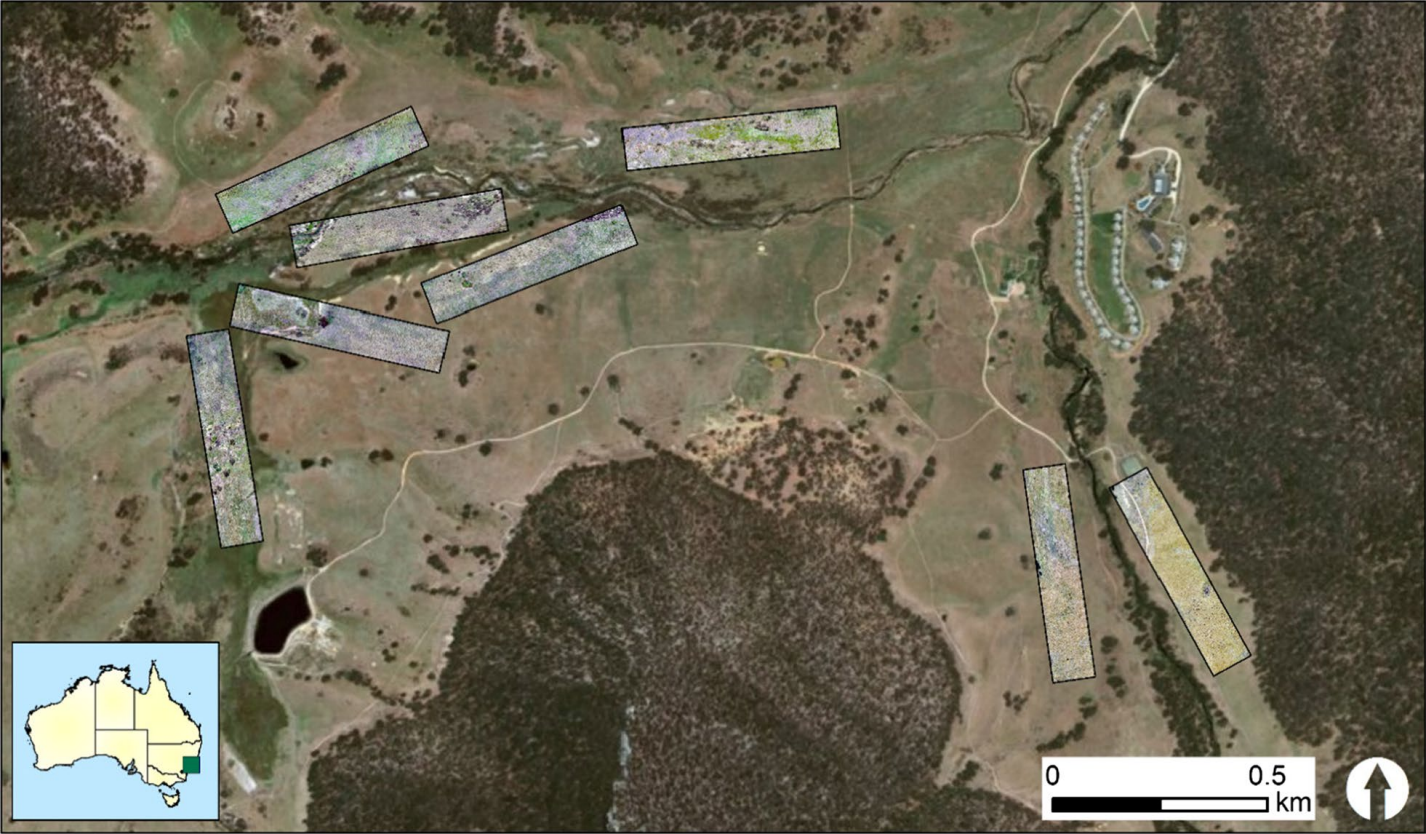
Bare-nosed wombat burrows were surveyed in transects in grassland communities of the Wolgan Valley, Australia. Transects were located on mostly flat areas adjacent to the Wolgan River that runs from west to east in the top of the image, and Carne Creek in the right of the image, flowing from the south. The base map is ArcGIS World imagery (Esri, DigitalGlobe, GeoEye, i-cubed, USDA FSA, USGS, AEX, Getmapping, Aerogrid, IGN, IGP, swisstopo, and the GIS User Community)

The Wolgan Valley (33° 15′S, 150° 10′E), New South Wales, Australia, was chosen as the study area to assess the effectiveness of a drone to survey wombat burrows. This site, previously a cattle station, has been managed as an ecotourism resort for the last decade and has large numbers of wombats and burrows [29, 30]. The sites surveyed within the 2830 ha property were situated in the river valley and grassland vegetation surrounding the Wolgan River and Carne Creek (Fig. 3).

We chose to survey grassland habitats at this study site. Grasslands adjacent to riparian zones have higher numbers of burrows [30, 37]. It also meant we could avoid trees (an animal ethics approval requirement A12180) and waterways. These areas also have higher densities of wombats, and are often valuable agricultural areas, so there is increased potential for wombats to come into conflict with humans and their activities in this habitat.

**Fig. 4:**
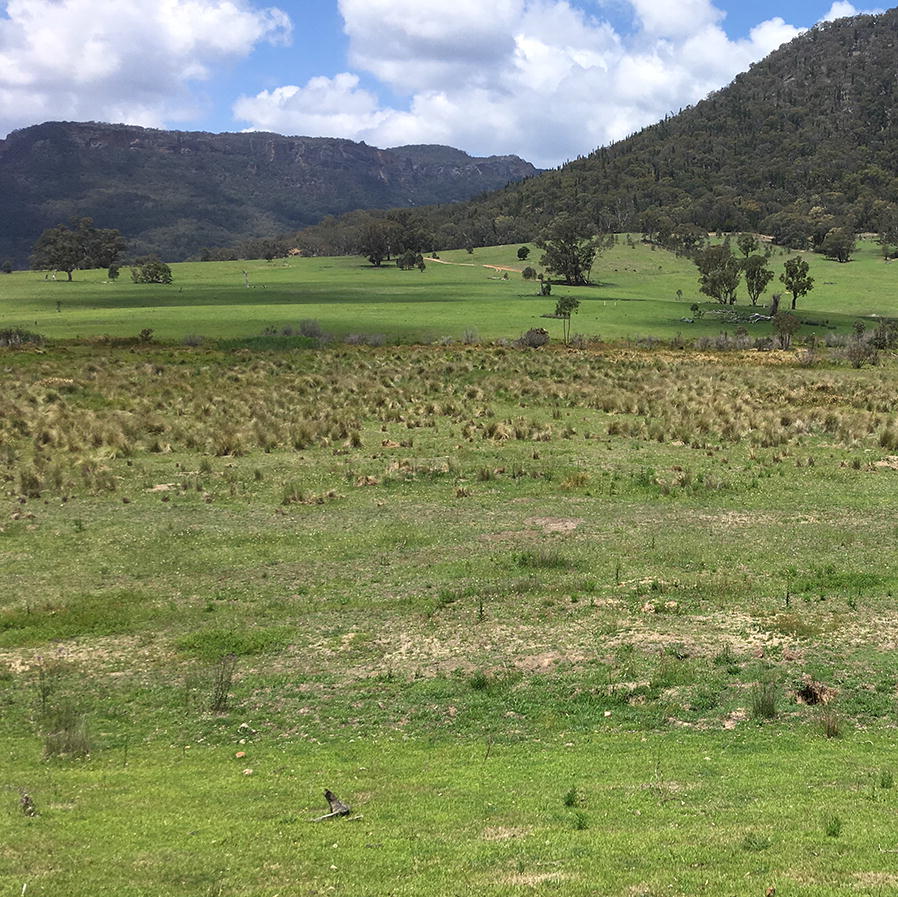
Grassland was the most common vegetation community in the study area and can be seen in this image in the foreground and rear slope leading up to the forest. Patches of tussock grassland were less extensive (centre of image) and were distributed on flat areas adjacent to the Wolgan River

Ground surveys and programmed drone flights for wombat burrows were conducted by observers in eight replicate 100 × 500 m (5 ha) transects (Fig. 3). Two different grassy communities occurred in transects, with very few shrubs. Trees were generally avoided as per our animal ethics approval, to minimise the potential for bird disturbance (Fig. 4). Grassland was the most widely distributed community within transects. Grasslands occurred on the flats and lower slopes of the valley adjacent to riparian areas and were composed of native and pasture species up to 15 cm high [20]. Patches of tussock grassland [28] occurred in particular sections of transects that were located on river flats, and averaged 66 cm in height. The transects contained no large water bodies, and at least some burrows (Fig. 4).

### Ground survey

The ground survey method to identify wombat burrows involved systematically walking transects and searching for wombat burrows [34]. A hand-held GPS (GPSmap 62s, Garmin, Australia) was used to record burrow coordinates when located. Three participants walked each transect and were spaced approximately 30 m from each other as they walked from one end of the 500 m transect to the other end of the transect. The 500 m length was estimated based on the area flown by the drone to ensure the entire site was covered. Burrows were recorded in a data sheet detailing the GPS co-ordinates, whether it was a burrow or not, and if the burrow was defined as active or inactive, as per Ostendorf et al. [31].

### Drone survey

A drone survey method was developed with the aim of capturing geographically referenced images from the drone for subsequent processing, to enable visual detection of wombat burrows by an analyst at the desktop. Firstly, the optimal height above the ground to capture images with the drone camera, in which burrows could be seen clearly, was determined. Clarity of burrows in images was greater at lower heights, but this had to be balanced against a corresponding reduction in the spatial extent of each survey, given finite drone battery life. All drone surveys were conducted using a Phantom 3 Professional drone (DJI, Shenzhen, China), which was operated manually with the DJI GO app (version 3.1.15), for this preliminary work. All images used in the study were captured using the original 12 megapixel RGB camera (model FC300X_3.6_4000x3000) supplied with the drone. This camera has a 6.16 mm wide × 4.62 mm high Sony EXMOR sensor with a focal length of 3.61 mm. The field of view is 94° with a 20 mm (35 mm format equivalent) f2.8 lens [9].

To ascertain the optimal height above the ground to fly the drone in surveys, the drone was positioned directly over a wombat burrow and images were captured at 20, 30, 40, 50, 60, 70 and 80 m above each burrow as the drone ascended vertically. This process was repeated for ten burrows with varying external characteristics. The resulting images were evaluated to assess the relative visibility of burrows at these heights. It was established that 40 m above the ground was the optimal height above ground at which to conduct drone surveys in the study area. The corresponding ground sampling distance for surveys was 2 cm.

Drone flights were conducted over each of the eight 100 × 500 m belt transects on the same days as ground surveys. The exact spatial extent of each survey transect was programmed as a grid (see Additional file 1) using the Pix4Dcapture app (Pix4D, Lausanne, Switzerland), which accommodates a range of user-specified flight settings. Images were taken with 80% front and side overlap, with the camera at 90° to the drone (directly facing the ground). The drone flew surveys at a speed of 5.2 m/s. Once each survey was set up using Pix4Dcapture, the operator initiated the flight, which ran automatically, but still required monitoring for irregularities and hazards during the survey. All drone flights were programmed such that the drone was visible to the operator during the flight, so that a flight could be aborted if, for example, a bird in flight approached the drone. Surveys were conducted at around midday to reduce the extent of shadows in resulting images. All transects were flown in dry weather, with no more than a little wind.

### Drone image processing

A separate, composite orthomosaic image of each survey transect was constructed from approximately 250 geotagged, drone-captured images per transect using Pix4Dmapper Pro photogrammetry software (V4.0.25, Pix4D, Lausanne, Switzerland). Automated production of each orthomosaic was a three-stage process (Pix4D 2017). Initially, specific features (keypoints) were extracted and matched in drone-captured images. Subsequent processing attributed 3D positions to keypoints to produce tie points, which were used in stage two to generate a densified point cloud. A digital surface model (DSM) was then created using the point cloud. The final orthomosaic was derived from the DSM and exported at 2 cm/pixel.

Orthomosaics were visually inspected using ArcMap (V10.5, Esri, California, United States) to detect burrows. The scale at which orthomosaics were scanned for burrows affected the capacity of the analyst to detect burrows in the images and required experimentation to establish. Orthomosaics were viewed at 1:100 scale; zooming into 1:40 for closer checks, and the point locations of individual burrows were marked manually in a separate layer of spatial data, which enabled comparison with plotted GPS points collected during ground surveys. Burrows were counted and the total number of burrows recorded for each transect.

To standardise burrow counting using drone and ground survey methods, burrow openings, and not burrows themselves, were recorded, as the underground extent and connectivity of burrows was uncertain. The occupancy status of burrows seen in orthomosaics was also estimated, i.e. whether the burrow was currently used by a wombat (potentially active) or not (inactive). The criterion used to classify a burrow as potentially active was that a patch of bare soil without any vegetation was evident at the burrow opening.

### Data analysis

To establish the extent to which the two survey methods were in agreement regarding their effectiveness in detecting burrows, paired t-tests were used initially to determine whether there was a difference in the number of burrows detected by the methods in transects [47]. When the methods were found to differ in this performance measure, the magnitude of the difference was calculated along with corresponding 95% confidence intervals. All statistical analyses for this study were conducted in R version 3.5.1 [32]. Because the structural and compositional differences between grassland and tussock grassland were expected to influence the effectiveness of using a drone to survey wombat burrows, analyses were conducted separately for the two communities. Tussock grassland was not present in two transects, so those transects were excluded from the t-test for that community. The distribution of the differences in numbers of burrows detected exclusively by each method in transects was approximately normal. We did not necessarily expect one method to out-perform the other as described above, so two-tailed t-tests were conducted.

To assess whether estimates of the occupancy status (active/inactive) of burrows were equivalent for drone survey and ground survey, McNemar’s test for comparing dependent proportions was used for burrows in the grassland community [1]. McNemar’s test was used to identify any systematic effects, or potential method bias in classification of occupancy status of burrows [47]. An exact binomial test was substituted for McNemar’s test for burrows in tussock grassland due to small sample size [1]. The data used for these tests was restricted to individual burrows detected by both survey methods. Individual burrows that had been assigned the same occupancy status classification by the two survey methods represented agreement between the methods. The number of these burrows was expressed as a percentage of the total number of burrows detected by both survey methods.

## Supplementary information

**Additional file 1.** Drone flights were programmed and conducted using the Pix4Dcapture app (V4.5.0) installed on an iPad (V11.4.1). This image is a view of the flight plan for one of the transects as seen in Pix4Dcapture. Parallel lines in the image show the path that was flown by the drone while surveying 100 × 500 m transects.

## Data Availability

The datasets for the current study are available from the corresponding author on reasonable request.

## Abbreviations

GIS: Geographic Information System
GPS: global positioning system
