# Supplementary material for: Mapping out bare-nosed wombat (Vombatus ursinus) burrows with the use of a drone
Source: BMC Ecol. 2019 Sep 18;19:39. doi: 10.1186/s12898-019-0257-5 (PMC6749681; doi:10.1186/s12898-019-0257-5)

Additional file 1. Drone flights were programmed and conducted using the Pix4Dcapture app (V4.5.0) installed on an iPad (V11.4.1). This image is a view of the flight plan for one of the transects as seen in Pix4Dcapture. Parallel lines in the image show the path that was flown by the drone while surveying 100 x 500 m transects.

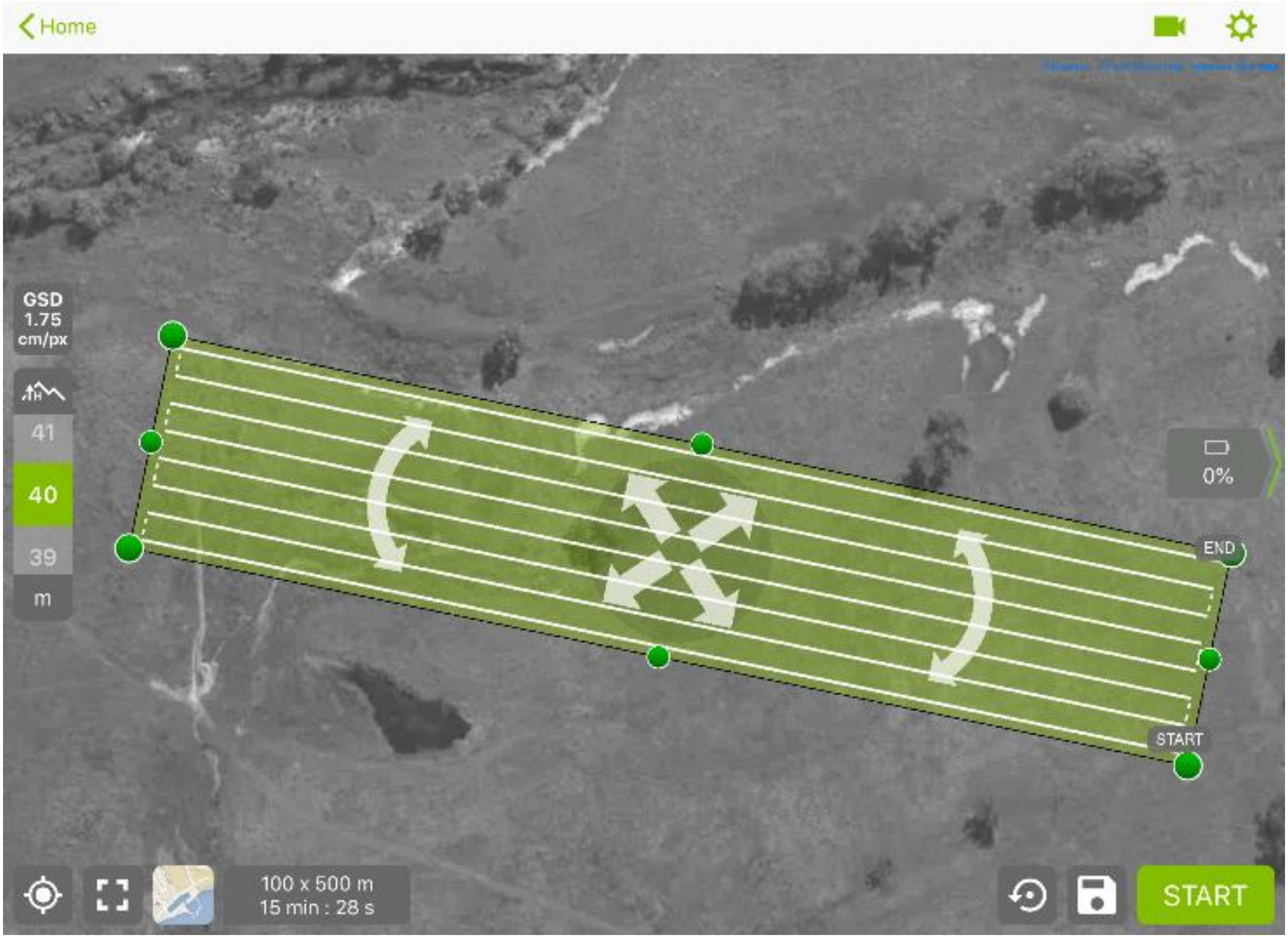

Supplement: Supplementary file 1 — Additional file 1. Drone flights were programmed and conducted using the Pix4Dcapture app (V4.5.0) installed on an iPad (V11.4.1). This image is a view of the flight plan for one of the transects as seen in Pix4Dcapture. Parallel lines in the image show the path that was flown by the drone while surveying 100 × 500 m transects. [file 12898_2019_257_MOESM1_ESM.pdf]
